# IDENTIFYING THE RELATIONSHIP BETWEEN ALZHEIMER’S DISEASE PREVALENCE AND CURRENT SERVICE USE IN OHIO’S 88 COUNTIES

**DOI:** 10.1093/geroni/igad104.3409

**Published:** 2023-12-21

**Authors:** Matt Nelson, Dr Heather Menne

**Affiliations:** Miami University, Oxford, Ohio, United States; Miami University, Oxford, Ohio, United States

## Abstract

Recently released data reveals that over 235,000 people over the age of 65 have Alzheimer’s disease and related dementia (ADRD) in Ohio. Using 2020 data from the National Center for Health Statistics, county-level specific ADRD prevalence estimates for Ohio’s 88 counties were also prepared (Range: 9.4-13.4 per 1000 individuals 65+). Researchers, practitioners, and policy-makers can use these estimates to better understand the reach and impact of ADRD in local communities. Nationally and in Ohio, care and support for older adults and specifically older adults with ADRD typically falls to family caregivers, home- and community-based service (HCBS) providers, and residential care providers such as nursing homes. This study pairs the county-level ADRD prevalence data with county-level data on available services (e.g., Memory Care Units, nursing home beds) and service users (e.g., HCBS users). The 10 counties in Ohio with the highest prevalence rates are home to nearly 60% of the 65+ population statewide. Additionally, these counties had a significantly higher proportion of older Black/Hispanic individuals than the other counties (10.8 versus 2.9). When looking at capacity, the top 10 counties contained just 12% of all nursing home beds, but 54% of nursing homes memory care units for the entire state. When considering service use, these 10 counties served a smaller percentage of the state’s HCBS users (13%). For Ohio and other states, it is important to explore available services and current service usage when faced with the increasing estimates of people living with ADRD.

